# Identification of serpins specific for activated protein C using a lysate-based screening assay

**DOI:** 10.1038/s41598-018-27067-z

**Published:** 2018-06-08

**Authors:** Stéphanie G. I. Polderdijk, James A. Huntington

**Affiliations:** University of Cambridge, Department of Haematology, Cambridge Institute for Medical Research, Wellcome Trust/MRC Building, Hills Road, Cambridge, CB2 0XY United Kingdom

## Abstract

Activated protein C (APC) is a powerful anticoagulant enzyme that proteolytically inactivates the cofactors of the Xase and prothrombinase complexes, factors VIIIa and Va. A common mutation in factor V, fV_Leiden_, confers resistance to APC leading to an increased risk of thrombosis in the normal population. However, when coinherited with haemophilia, fV_Leiden_ reduces bleeding severity, suggesting that inhibition of APC may be a useful strategy for treatment of haemophilia. We previously reported on serpins that were rationally designed for improved specificity for APC over other coagulation serine proteases. Based on structural differences in the substrate binding pockets to either side of the P1 Arg, we mutated the P2 and P1′ residues to Lys. Although this approach achieved APC specificity, it resulted in a reduction in the rate of APC inhibition relative to the parent containing only the P1 Arg. Here we conduct site-specific random mutagenesis at the P2 and P1′ positions to determine if improvements could be made in the rate of APC inhibition. In addition to our original Lys mutations, we found that Arg and Gln also confer specificity for APC. However, in all cases specificity for APC resulted in a reduction in inhibition rate.

## Introduction

**Figure 1:**
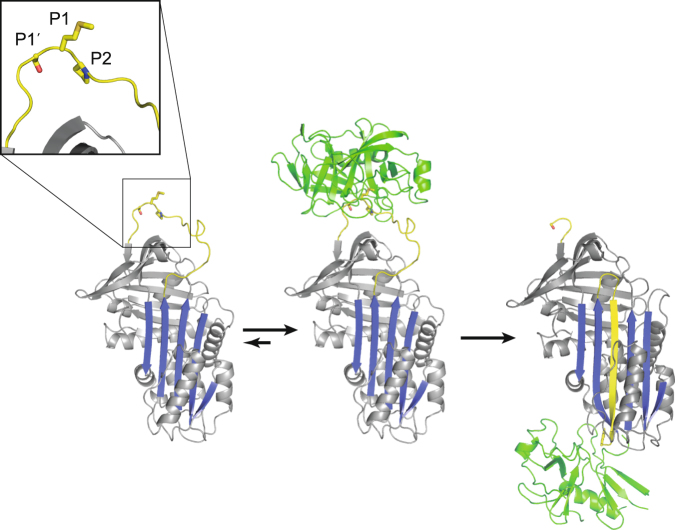
The serpin mechanism of protease inhibition. In their native state, serpins have an exposed reactive centre loop (RCL, yellow) and a five-stranded β-sheet A (blue, left panel). The RCL contains the scissile P1-P1′ bond that is recognised as a substrate by serine proteases (green). The principal specificity-determining residue is P1, but residues to either side (P2 is N-terminal and P1′ is C-terminal) also contribute (close-up box). The RCL is bound in the active site of a cognate serine proteases as a substrate to form the reversible recognition or Michaelis complex (central panel). At the acyl-enzyme intermediate step of proteolysis, where an ester bond exists between the catalytic serine and the P1 residue of the serpin, the N-terminal portion of the RCL rapidly inserts into β-sheet A, flinging the protease to the opposite pole of the serpin and distorting the oxyanion hole, thus preventing deacylation (right panel). Serpin-protease complexes are thus covalent and irreversible.

The serpins are a family of serine protease inhibitors that utilise a conserved two-step suicide-substrate mechanism^1^ (Fig. 1). The rate-limiting and specificity-determining step is the formation of the recognition, or Michaelis complex, where the reactive centre loop (RCL) of the serpin is accommodated in the active site cleft of the protease in a substrate-like fashion. The protease then cleaves the scissile bond of the serpin (P1-P1′) triggering a large conformational rearrangement involving the incorporation of the RCL into β-sheet A and translocation of the protease to the opposite pole of the serpin. The protease is trapped in the final complex at the acyl-enzyme intermediate stage of proteolysis, with an ester bond between the catalytic Ser Oγ and the main chain C of the P1 residue^2^. The purpose of the conformational change is presumably to disrupt the structure of the protease, including the distension of the oxyanion hole, to prevent deacylation. The specificity of serpins for their target proteases is largely determined by the P1 composition, but residues to either side, in particular, P4, P2 and P1′, also play a role. In some cases, cofactors and exosites can also contribute^3,4^.

**Figure 2:**
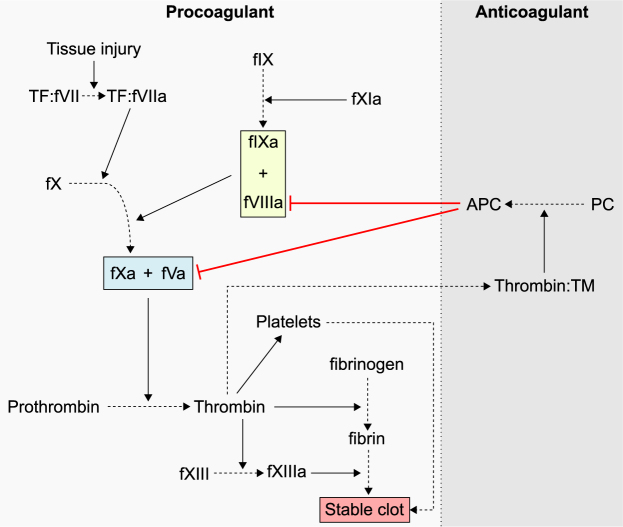
Simplified overview of the coagulation cascade. Tissue injury exposes tissue factor (TF) to fVIIa, thereby activating the extrinsic pathway of coagulation and producing fXa. Prothrombinase, composed of fXa and the cofactor fVa, converts prothrombin to thrombin. Thrombin starts a positive feedback loop allowing activation of more fV, as well as fVIII and fIX (through fXI). The intrinsic Xase complex (fIXa + fVIIIa) activates more fX. The resulting thrombin forms a stable clot by activating platelets and by cleavage of fibrinogen to fibrin. These procoagulant processes are balanced by anticoagulant pathways, such as the protein C pathway (right). Protein C (PC) is activated by thrombin bound to thrombomodulin (TM). Activated protein C (APC) cleaves and inactivates fVa and fVIIIa, shutting down the prothrombinase and Xase complexes (for a review of the coagulation cascade, see Hoffman and Monroe, 2007^36^). Solid arrows show activation reactions, red capped arrows show inhibition reactions.

Several serpins are present in the circulation, including α_1_-antitrypsin (α_1_AT, SERPINA1, also known as α_1_-proteinase inhibitor)^5^, antithrombin (SERPINC1)^6^, heparin cofactor II (SERPIND1)^7^, plasminogen activator inhibitor-1 (SERPINE1)^8^ and protein C inhibitor (SERPINA5)^9^, all of which contribute in some measure to the regulation of blood coagulation (haemostasis). Haemostasis is traditionally depicted as a cascade of protease activation events, where a small trigger leads to a burst of the effector protease thrombin (Fig. 2). Thrombin is a potent platelet agonist and is the only enzyme capable of converting fibrinogen into fibrin to form the meshwork that gives structure to clots^10–13^. Pro- and anticoagulant forces are finely balanced to ensure an appropriate response to injury (Fig. 2). Disturbances of this balance leads either to excessive thrombin generation, resulting in thrombosis, or insufficient thrombin generation, resulting in bleeding. Haemophilia refers to a family of bleeding disorders caused by defects or deficiencies in coagulation factor (f) VIII (haemophilia A), fIX (haemophilia B) or fXI (haemophilia C)^14,15^. The function of the factors missing in haemophilia is to produce a second burst of fXa, the protease component of the prothrombinase complex (fXa-fVa) and thereby contribute to the formation of thrombin.

The current standard of care in haemophilia is intravenous infusions of replacement factors, either prophylactically or on demand^16,17^. However, fVIII and fIX are occasionally recognised as foreign proteins, resulting in the development of inhibitory antibodies^18^. These ‘inhibitor’ patients are treated with so-called ‘bypassing’ agents, such as NovoSeven (recombinant fVIIa) and FEIBA (factor eight inhibitor bypassing activity)^19^. All currently approved therapies for haemophilia increase the concentration of procoagulant factors, however, it is also possible to rebalance haemostasis by reducing the concentration or activity of intrinsic anticoagulants^20–23^.

We recently employed a structure-based rational mutagenesis approach to develop a serpin specific for activated protein C (APC)^23^. APC acts as a powerful anticoagulant by proteolytically inactivating the cofactors of the intrinsic Xase and prothrombinase complexes, fVIIIa and fVa^24^. The modified serpin improved thrombin generation *in vitro* and rescued haemostasis in haemophilia B mouse models. We mutated the P2-P1′ region of α_1_AT from PMS to KRK to confer specificity for APC over fXa and thrombin, with rate constants of 15,000, 120 M^−1^·s^−1^ and not detectable, respectively. However, the price for specificity was a 7-fold reduction in the rate of APC inhibition relative to the parent molecule with only the P1 mutation^23^.

To determine if other sets of residues flanking the P1 Arg on the α_1_AT background might have similar APC specificity with improved potency, we developed a bacterial lysate-based screening assay that allows for the medium-throughput screening of the P2 and P1′ positions individually and in combination. Using this assay, we identified two additional combinations with similar specificity for APC over thrombin and fXa, but with no improvement in potency. We conclude that the variant designed by rational mutagenesis (KRK) is not unique in its specificity for APC, but is nevertheless an optimal combination for the P2-P1′ region on the α_1_AT template.

## Results

### Assay validation

**Figure 3:**
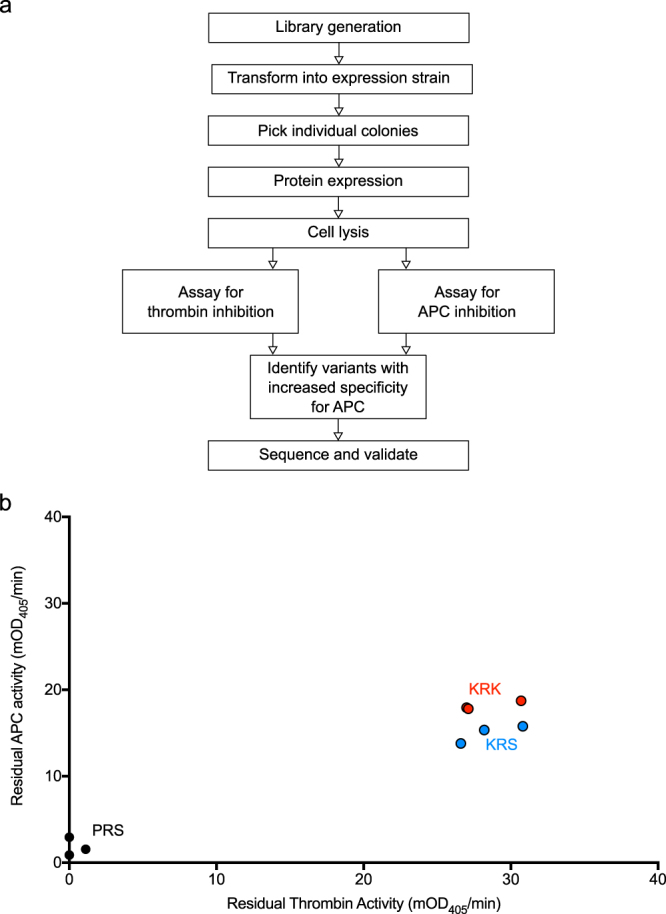
Assay validation using α_1_AT variants with known inhibitory properties. (**a**) Flow chart of the bacterial lysate assay. (**b**) Results from the initial validation assay. Residual protease activity after incubation of the protease with bacterial lysates after expression of α_1_AT variants with known inhibitory properties. PRS α_1_AT (P2 Pro, P1 Arg and P1′ Ser) is shown in black; KRS α_1_AT in blue and KRK α_1_AT in red. Each point is derived from an individual colony, grown, expressed, lysed and assayed separately.

Variants of α_1_AT with known inhibition rates towards thrombin and APC^23^ were used to assess the sensitivity of the bacterial lysate screening method, outlined in Fig. 3a. Inhibition rates for these variants were determined previously and are given in Table 1 for reference. Plasmids containing the coding sequences for PRS, KRS and KRK α_1_AT were transformed into Rosetta2(DE3)pLysS cells and grown in 96-well deep well blocks. After induction with IPTG and an overnight incubation at 25 °C, cells were harvested by centrifugation and lysed using lysis buffer and freeze-thaw cycles. After clarification of the lysate by centrifugation, inhibition of thrombin and APC was tested by incubation of a sample of the lysate with the respective protease. After 20 min for APC and 1 h for thrombin, chromogenic substrates were added and residual protease activity determined. The results are plotted as residual protease activity for thrombin and APC on the ordinate and abscissa, respectively (Fig. 3b). A decrease in the residual protease activity for one variant relative to another variant is indicative of an improved inhibition rate.

**Table 1:** Inhibition rates and aPTT results for α_1_AT variants.

Variants	*k*_2_ (M^−1^·s^−1^)			aPTT (s) NR = 60.3 ± 3.4
	APC	Thrombin	fXa	
KRS α_1_AT	(6.48 ± 0.71) × 10^4^*	(5.10 ± 0.28) × 10^1^*	(3.93 ± 0.31) × 10^3^*	107.2 ± 3.7
PRK α_1_AT	(9.57 ± 1.37) × 10^4^*	(1.74 ± 0.17) × 10^2^*	(4.89 ± 0.16) × 10^3^*	228.1 ± 6.6
RRS α_1_AT	(6.11 ± 0.63) × 10^4^	(4.24 ± 0.24) × 10^1^	(4.79 ± 0.58) × 10^3^	111.6
PRR α_1_AT	(1.32 ± 0.13) × 10^5^	(6.82 ± 0.68) × 10^2^	ND	287
PRE α_1_AT	(2.99 ± 0.29) × 10^3^	(1.47 ± 0.15) × 10^2^	ND	84
TRN α_1_AT	(6.24 ± 0.25) × 10^4^	(2.74 ± 0.47) × 10^2^	ND	>300
TRY α_1_AT	(5.70 ± 0.83) × 10^3^	(2.34 ± 0.14) × 10^1^	ND	185.4
QRK α_1_AT	(3.34 ± 0.64) × 10^4^	(3.81 ± 1.32) × 10^0^	(1.08 ± 0.15) × 10^3^	111.5
KRH α_1_AT	(2.88 ± 0.30) × 10^4^	No detectable inhibition	(6.22 ± 0.40) × 10^2^	77.8
KRN α_1_AT	(3.78 ± 0.25) × 10^4^	(1.50 ± 0.26) × 10^1^	(9.11 ± 1.30) × 10^2^	81.9
RRC α_1_AT	(2.46 ± 0.22) × 10^4^	(3.36 ± 0.94) × 10^1^	ND	ND
PRS α_1_AT	(1.08 ± 0.071) × 10^5^*	(2.93 ± 0.18) × 10^5^*	(4.13 ± 0.24) × 10^4^*	>300*
KRK α_1_AT	(1.51 ± 0.17) × 10^4^*	No detectable inhibition*	(1.16 ± 0.10) × 10^2^*	62.1 ± 4.2*

NR is the value for the buffer only control. The values marked with * are from our previous study^23^ and are shown for comparison. ND indicates values that were not determined.

PRS α_1_AT showed little residual thrombin or APC activity under the conditions of the assay, consistent with our previously measured rate constants of ~300,000 and ~100,000 M^−1^·s^−1^, respectively (Table 1). KRS α_1_AT, which inhibits thrombin very slowly, showed high residual thrombin activity, as did KRK α_1_AT, which has no detectable thrombin inhibition. The rate constants of APC inhibition for KRS and KRK are ~65,000 and ~15,000 M^−1^·s^−1^. Consistent with this, KRS α_1_AT showed lower residual APC activity than KRK α_1_AT in the screening assay (Fig. 3b). There is some variability between lysates derived from separate colonies containing the same sequence. This variability is probably attributed to differences in growth/expression resulting in variable levels of inhibitor, even in colonies expressing the same variant. However, overall these results suggest that the assay design can identify specific APC inhibitors and may be able to rank them with respect to potency.

### Screening of random P2, P1′ and P2P1′ libraries against thrombin and APC

**Figure 4:**
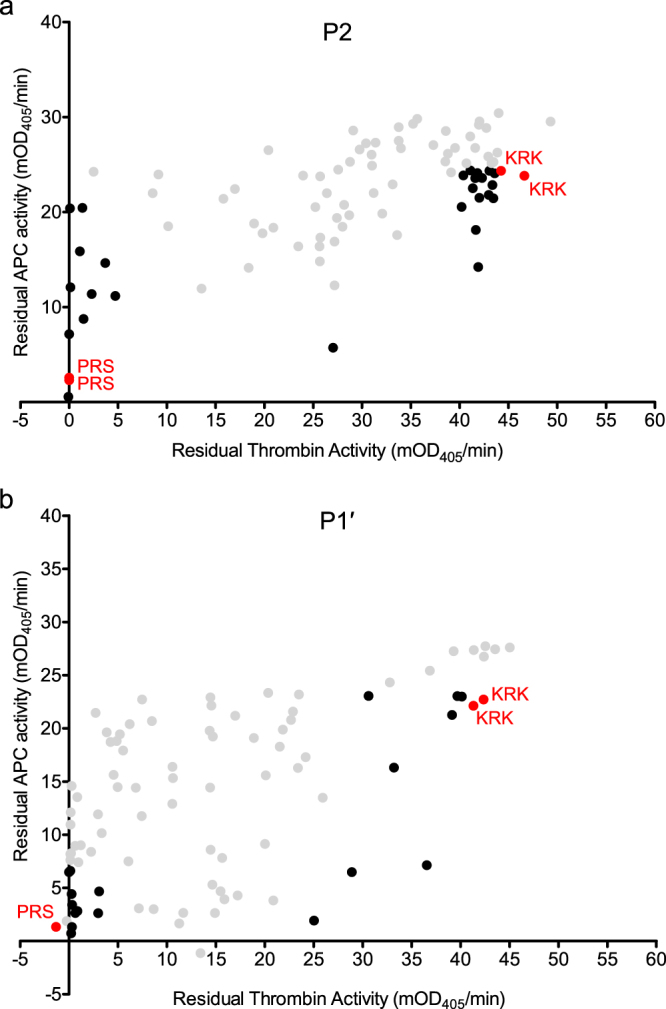
Screening of α_1_AT PRS libraries randomised at P2 or P1 ´. Plots show residual APC activity vs. residual thrombin activity after incubation with the respective bacterial lysate. Variants picked for rescreening are shown in black. Controls are shown in red. (**a**) Screening results for the library randomised at P2. (**b**) Screening results for the library randomised at P1 ´.

**Figure 5:**
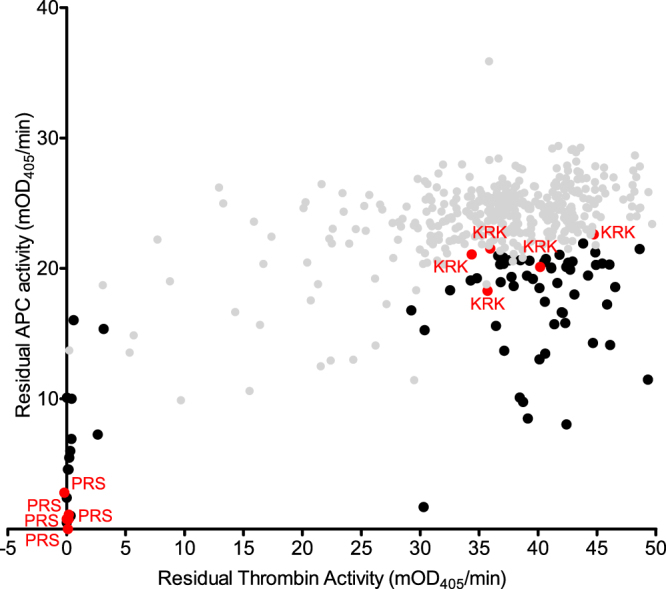
Screening of α_1_AT PRS libraries, randomised at P2 and P1′. Plot shows residual APC activity vs. residual thrombin activity after incubation with the bacterial lysates. Variants picked for rescreening are shown in black. Controls are shown in red.

**Figure 6:**
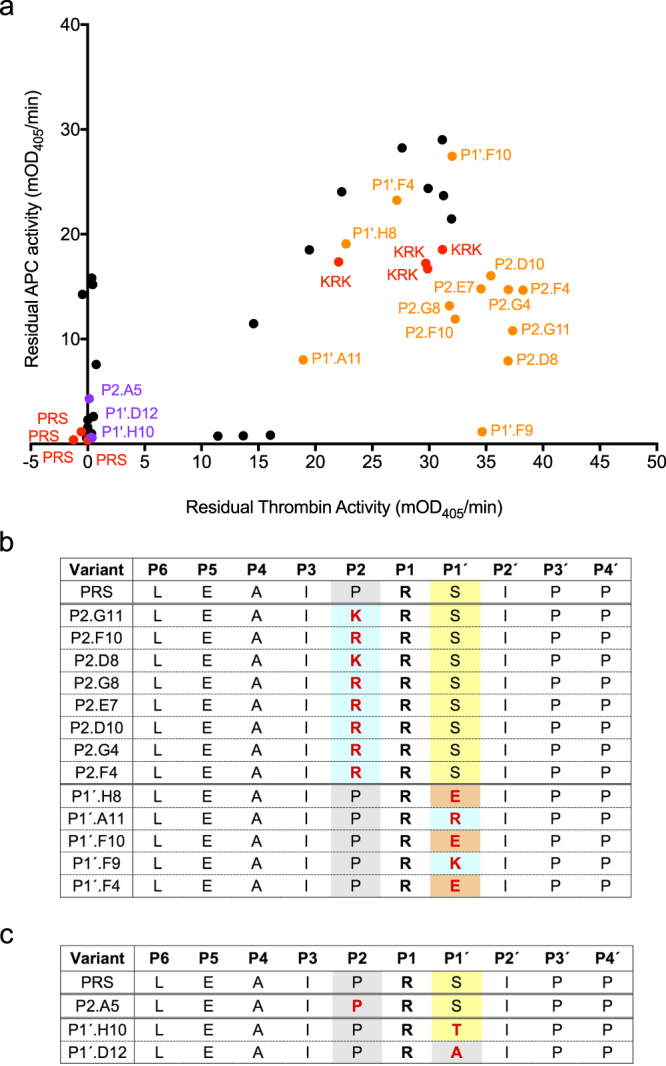
Validation screen of α_1_AT variants mutated at either P2 or P1′. (**a**) The validation assay is of a subset of variants picked from the initial screen (shown in black in Fig. 4). Residual protease activity after incubation of the protease with bacterial lysates after expression of α_1_AT variant libraries is shown. Control cultures, expressing PRS and KRK α_1_AT are shown in red, cultures of interest showing low residual APC activity and high residual thrombin activity that were sequenced are shown in orange. All variants shown in orange were further characterised (data in Table 1) or have previously been characterised^23^. Variants that were nonspecific inhibitors of both thrombin and APC and were sequenced for comparison are shown in purple. The prefix P2 or P1′ indicates whether this variant was originally from the P2 or P1′ variant library. (**b**) P6-P4′ sequences of potential APC-specific variants (shown in orange in (**a**)). The P1 R residue is shown in bold. Residues that were varied in the respective library are shown in red. P2 and P1′ residues are coloured according to properties with positively charged residues in blue, small apolar residues in grey, small polar residues in yellow and negatively charged residues in orange. (**c**) P6-P4′ sequences of nonspecific α_1_AT variants (shown in (**a**) in purple). Colouring as in (**b**).

**Figure 7:**
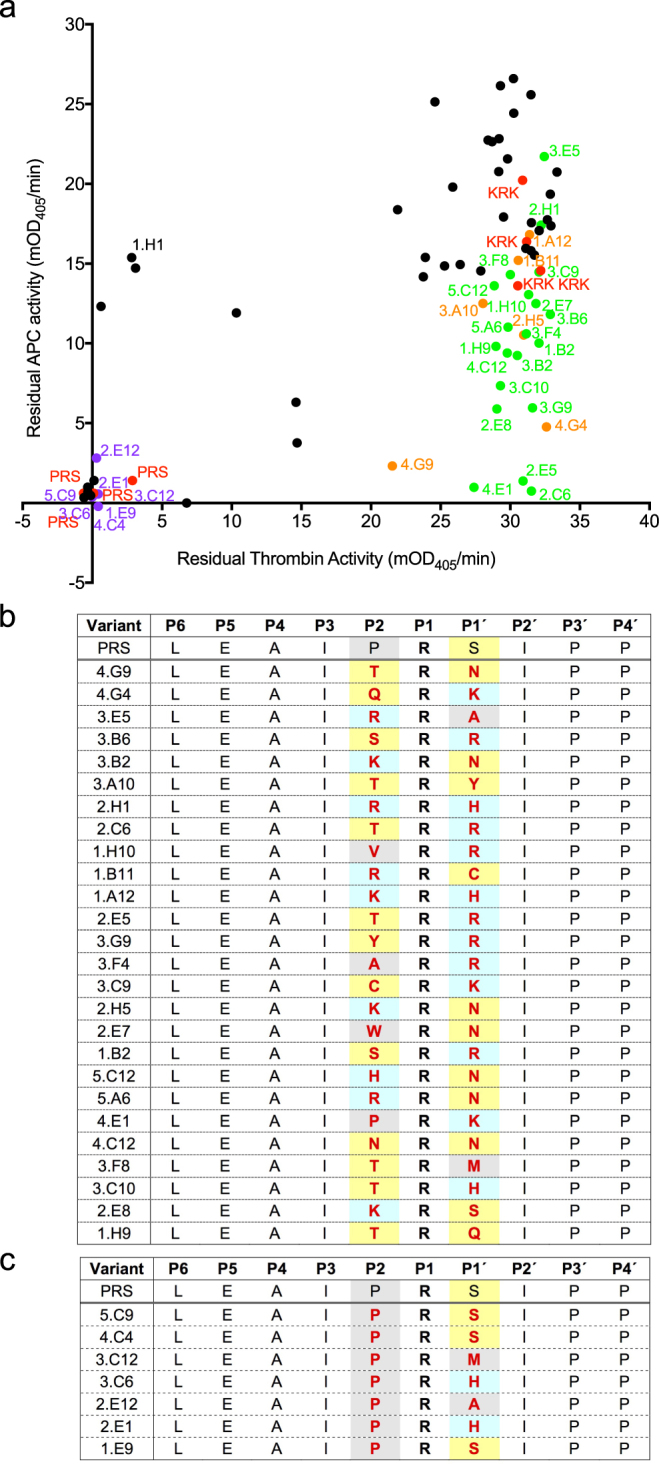
Validation screen of α_1_AT variants mutated at both P2 and P1′. (**a**) The validation assay is of a subset of variants picked from the initial screen (shown in Fig. 5). Residual protease activity after incubation of the protease with bacterial lysates after expression of α_1_AT variant libraries is shown. Control cultures, expressing PRS and KRK α_1_AT are shown in red, cultures of interest showing low residual APC activity and high residual thrombin activity that were sequenced are shown in green. Of these, a subset, which was not previously studied was characterised further (data in Table 1) and these are shown in orange. Variants that were nonspecific inhibitors of both thrombin and APC and were sequenced for comparison are shown in purple. (**b**) P6-P4′ sequences of potentially APC-specific variants (shown in green and orange in (**a**)). The P1 R residue is shown in bold. Residues that were varied in the respective library are shown in red. P2 and P1′ residues are coloured according to properties with positively charged residues in blue, small apolar residues in grey, small polar residues in yellow and negatively charged residues in orange. The template PRS α_1_AT is shown for comparison. (**c**) P6-P4′ sequences of nonspecific α_1_AT variants (shown in purple in (**a**)). Sequences are coloured as in (**b**).

Randomised libraries were generated using a degenerate NNS codon for varying P2 or P1′ individually. The “small-intelligent” library approach^25^ was used to generate the combined P2P1′ library. Mutants from these libraries were expressed and cell lysates assayed for protease inhibition in a 96-well format using the endpoint assay described above. Results for the screening of 88 colonies each for the individual libraries and 460 colonies for the double mutant library are shown in Figs 4 and 5. Colonies with high residual thrombin activity (low thrombin inhibition) and low residual APC activity (high APC inhibition) were chosen for further evaluation, and some colonies that showed inhibition of both thrombin and APC were also picked for comparison. The colonies selected from the initial screens were regrown, re-expressed and the lysates assayed for protease inhibition to validate the initial hits. Colonies that showed repeatable residual activities were then sequenced. The results from the validation assay are shown in Fig. 6a for the single variant libraries and Fig. 7a for the P2P1′ library, with sequences shown in Figs 6b and 7b, respectively.

APC-specific variants from the single P2 or P1′ libraries showed little sequence variation. Preferred P2 residues were either K or R, and P1′ residues were K, R or E (Fig. 5b). From our rational design study, we knew K and R would improve APC specificity, but P1′ E was surprising because the S1′ subsite of APC is negatively charged. Serpins that showed good inhibition of both APC and thrombin were identical or very similar to the PRS parent, with P2 always proline and a small P1′ of either S, T or A.

APC-specific variants from the P2P1′ randomised library showed a wider range of sequences, indicating a certain degree of cooperativity between these positions (Fig. 7b). While many variants had K and R residues, polar residues such as H, Q and N, and large hydrophobics such as M, W and Y were also found. Non-specific variants all had a P2 proline, consistent with previous reports that this is a preferred residue for both thrombin and APC^26,27^, and the P1′ residues included the serine present in the parent molecule, as well as A, M and H.

### Kinetic characterisation of hits

A panel of novel variants distinct from the previously identified KRK variant was picked for purification and further characterisation. Rates of inhibition of APC and thrombin were determined for PRR, PRE, TRN, TRY, QRK, KRH, KRN and RRC (Table 1). We previously reported that KRK α_1_AT inhibited APC at a rate of ~15,000 M^−1^·s^−1^ and showed no detectable inhibition of thrombin^23^. Out of the variants identified in the screening assay, RRS, PRR, TRN, QRK, KRH, KRN and RRC all inhibited APC more efficiently than KRK (Table 1). Rate constants of thrombin inhibition were very low for RRS, TRY, QRK, KRH, KRN and RRC (Table 1). These results indicated that the screening assay was capable of selecting serpins specific for APC over thrombin, and was successful in identifying several new candidate APC inhibitors.

The effect of the mutants on clotting time in an aPTT assay was evaluated (Table 1) to screen for inhibition of other procoagulant proteases, such as fIXa, fXa and fXIa. At a concentration of 5 μM, KRK does not increase the aPTT^23^. In contrast, all of the other variants resulted in an increase in aPTT: PRE, KRH and KRN caused a 30–40% increase; RRS and QRK almost doubled the aPTT; TRY tripled the aPTT; PRR resulted in a 5-fold increase; and addition of TRN rendered the aPTT unclottable by the end of the 300 s experiment. Based on the criteria of high rate of APC inhibition, low rate of thrombin inhibition and modest increase in aPTT, four mutants, RRS, QRK, KRH and KRN were selected for further evaluation.

Kinetics of fXa inhibition were determined for the selected variants, and rate constants are shown in Table 1. RRS was found to be a good inhibitor of fXa, with a rate constant of about 5,000 M^−1^ s^−1^, probably accounting for its 2-fold increase in aPTT. QRK, KRH and KRN were moderate inhibitors of fXa, with rate constants ranging from 600–1,000 M^−1^ s^−1^, between 6–9-fold greater than the rate of fXa inhibition by KRK α_1_AT. It is unclear if this level of fXa inhibition would be acceptable in a haemophilia model, especially since their rate of APC inhibition is only 2-fold improved over KRK. These experiments indicated the need to include an additional counter-screen of random variants against fXa.

### Screening of libraries against fXa

**Figure 8:**
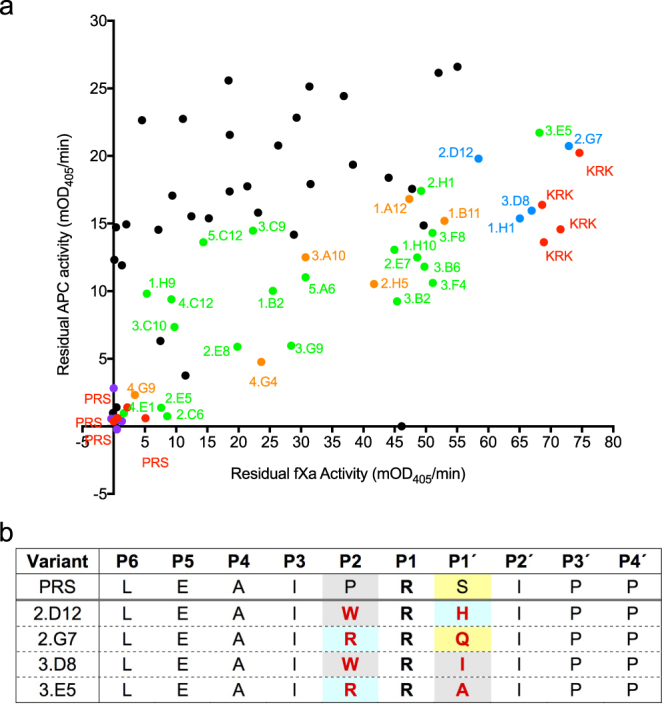
Counter-screen for fXa inhibition identifies potential candidate serpins. Screening of the variants shown in Fig. 7 against fXa. (**a**) Comparison of the residual APC activity (from Fig. 7) plotted against residual fXa activity. Data points are coloured as in Fig. 7 except that variants not previously sequenced and showing low fXa inhibition (high residual fXa activity) and substantial APC inhibition (reduced residual APC activity) that were sequenced as a result of this assay are shown in blue. (**b**) P6-P4′ sequences of the variants showing α_1_AT variants potentially specific for APC over fXa and thrombin. These include three of the four variants labelled in blue in (**a**) and one previously sequenced variant from Fig. 7. The template PRS α_1_AT is shown for comparison.

Lysates from the P2P1′ validation screen (Fig. 7) were rescreened against APC and fXa, and the results are shown in Fig. 8. This screen appeared to not identify any mutants with an equally low fXa inhibition and greater APC inhibition than KRK α_1_AT (Fig. 8a). Nevertheless, the screen identified a few variants that were not sequenced previously that had activities similar to KRK (Fig. 8b). Mutant 1.H1, was excluded because it showed substantial thrombin inhibition (indicated on Fig. 7a).

The newly sequenced mutants had either R or W at P2 and H, Q, I or A at P1′ (Fig. 8b). These variants were expressed, purified and characterised by running a single concentration inhibition rate measurement against thrombin and fXa to determine preliminary inhibition rate constants. From these single measurements, rate constants for thrombin inhibition were estimated to be 21 M^−1^·s^−1^ for RRA, 11 M^−1^·s^−1^ for RRQ, 50 M^−1^·s^−1^ for WRH and 3 M^−1^·s^−1^ for WRI. For fXa inhibition, these rates were estimated to be 4,100 M^−1^·s^−1^ for RRA, 140 M^−1^·s^−1^ for RRQ, 4,900 M^−1^·s^−1^ for WRH and 4,800 M^−1^·s^−1^ for WRI. While all variants had low thrombin inhibition, fXa inhibition was substantial for WRI, WRH and RRA, with only RRQ exhibiting both low fXa and thrombin inhibition. Hence, the fXa screen appeared to be less sensitive than the thrombin and APC screens. Perhaps longer incubation times with fXa could improve upon this in the future. RRQ was considered potentially interesting and was characterised further.

### Characterisation of newly identified hits

Based on the results described above, R and K mutations were largely equivalent in terms of specificity in the P2 and P1′ positions. We therefore constructed a KRQ variant, in addition to the RRQ variant, for kinetics and clotting assays (Table 2). Both variants had extremely low rates of thrombin inhibition, and fXa inhibition was similar to the rate previously measured for KRK α_1_AT (compare Tables 1 and 2). In addition, neither variant prolonged the aPTT, suggesting negligible inhibition of other coagulant proteases. However, the rate of APC inhibition was slightly lower than that of our previously identified lead, KRK α_1_AT. Therefore, although random mutagenesis successfully identified new APC-specific variants with similar properties to KRK α_1_AT, it appears that specificity conferred through mutations at P2 and P1′ comes with a cost to inhibitory efficiency.

**Table 2:** Inhibition rates and aPTT results for best variants.

Variants	*k*_2_ (M^−1^·s^−1^)			aPTT (s) NR = 49.3 ± 2.5
	APC	Thrombin	fXa	
RRQ α_1_AT	(8.3 ± 1.1) × 10^3^	(5.4 ± 1.1) × 10^0^	(1.3 ± 0.1) × 10^2^	55.1 ± 2.8
KRQ α_1_AT	(9.0 ± 0.7) × 10^3^	(2.9 ± 1.5) × 10^0^	(1.7 ± 0.1) × 10^2^	53.9 ± 3.0

NR is the value for the buffer only control.

## Discussion

Serpins have previously been the target of rational engineering as well as randomisation to alter inhibitory properties. One example is the effort to engineer α_1_AT for improved inhibition of thrombin. Hopkins *et al*.^28^ replaced the P7-P3′ residues of α_1_AT with the corresponding residues in antithrombin or heparin cofactor II^29^. Attempts were made to improve these inhibitors by random mutagenesis and screening approaches, using either a bacterial lysate screening approach^30^ or a phage display method^31^. Similar efforts were undertaken to generate serpins specific for furin^32^, kallikrein 2 and kallikrein 14^33^, but no counter-selection against other proteases was performed as part of the screening.

We have shown here that a bacterial lysate-based protease inhibition assay can be used to select protease-specific serpins from selectively randomised libraries using both positive and negative screening. We recognise that the method has some shortcomings that subsequent users may wish to address. Firstly, we chose to vary only RCL positions that we had previously determined to be critical for specificity for APC over thrombin. Ideally, we would want to vary additional positions, to identify cumulative and cooperative effects. However, while screening lysates allows higher throughput than characterisation of purified variants, the amount of manual handling involved in the protocol limits the number of positions one can test. One potential for improving the throughput would be to reduce the assay volumes to allow use of 384-well plates using a robotics setup, which would dramatically increase the number of positions that could be varied simultaneously. Additionally, the screening method as described here is not adjusted for serpin concentration in each lysate. Mutations can cause unexpected changed in ability of a protein to fold, and clones vary in growth and expression levels. ELISA-based methods could be used to quantitate the amount of serpin in each lysate and final assay volumes adjusted accordingly to account for expression differences. However, the power of this method is its ease, and adjusting the lysate volume for each reaction would be overly cumbersome and impact the throughput. Altered levels of expression are also partially accounted for by a selection based on the ratio of inhibition of target and non-target proteases.

This study was undertaken to determine if potency against APC could be improved while maintaining the degree of selectivity against other coagulation proteases obtained through structure-based rational mutagenesis. The mutations found by the screens described here are consistent with our previous structure-based design results^23^. We showed that positively charged residues at P2 and P1′ promote specificity for APC over thrombin and fXa, a result confirmed by the R and K mutations found in both the random single and double mutant screens. Other amino acids with large side chains were also able to confer specificity over thrombin. In contrast, all non-specific variants sequenced contained a P2 proline, consistent with the known preference for thrombin^27^.

Factor Xa substrate preferences are less well defined than those of thrombin. Previous work suggested a paradoxical preference for glycine or large hydrophobics at P2^34^. Therefore, the results from the fXa screen were initially surprising when a P2 tryptophan appeared to be detrimental to fXa. However, further characterisation of the P2W variants found that they maintained substantial fXa inhibitory activity. Only positively charged residues at P2 *and* P1′ could introduce specificity for APC over fXa (with the exception of Q at P1′), indicating that the positively charged residues we originally used were ideal at these positions.

Although we were unable to identify α_1_AT variants with improved potency for APC while maintaining specificity, we were successful in identifying two additional variants with profiles similar to KRK. The additional variants both had a Q at P1′ that is isosteric to K, indicating that charge is less important than size at that position. Future studies targeting additional residues such as P4, P3, P2′ and P3′ could be carried out to potentially improve APC inhibition while maintaining specificity.

In summary, although there are certain limitations to consider, the results described here support the utility of our assay in identifying protease-specific serpins from large pools of randomly generated RCL mutants using both positive and negative selection.

## Materials and Methods

### Materials

Human plasma proteases were purchased from Hematologic Technologies. Chromogenic substrates were from Chromogenix.

### α_1_AT library generation and expression

α_1_AT (SERPINA1) variant libraries were generated by site-directed mutagenesis on the pETSUMO full-length PRS α_1_AT background with the additional E1S and C232S mutations described previously^23^. The P2 and P1′ mutant libraries were made by site-directed mutagenesis using a degenerate primer containing an NNS codon at either the P2 or P1′ position. For the P2P1′ library a mixture of primers with different degenerate codons as described by Tang *et al*.^25^ was used to allow generation of a library with only one codon per amino acid, reducing the redundancy of the library. The mutagenesis reactions were transformed into α-Select Gold competent cells (Bioline) for amplification and estimation of library size and plated on LB plates containing kanamycin. From these plates, ~1000 colonies were scraped for the single mutant libraries and ~3000 for the double mutant libraries into liquid LB medium with kanamycin. Pooled plasmid libraries were extracted using standard DNA extraction methods and sequenced at Source Bioscience (Cambridge) using Sanger sequencing to verify randomisation of the target codon. For expression of the library, the plasmid pool was subsequently transformed into Rosetta2(DE3)pLysS cells (Novagen).

### Library screening

Colonies of Rosetta2(DE3) pLysS cells (Novagen) transformed with α_1_AT libraries, as described above, were picked into 2xTY with kanamycin (30 μg/ml) and chloramphenicol (34 μg/ml) in sterile, deep-well 96-well blocks and grown overnight at 37 °C with shaking at 250 rpm. For single position variant libraries 88 variants were picked, and for the double variant library 460 variants were picked for determination of protease inhibitory activity. Each 96-well plate included PRS α_1_AT and KRK α_1_AT controls. Each colony was additionally streaked onto LB agar plates containing 30 μg/ml kanamycin for archiving. Overnight cultures were diluted 40-fold into 1 ml 2xTY with kanamycin (30 μg/ml) and chloramphenicol (34 μg/ml) in sterile, deep-well 96-well plates and grown for 7 h at 37 °C with shaking at 250 rpm. Cultures were induced with 0.6 mM IPTG and proteins expressed overnight at 25 °C. Cells were harvested by centrifugation, pellets resuspended in 200 μl lysis buffer (20 mM Tris pH 7.4, 150 mM NaCl, 1 mg/ml lysozyme, 5 U/ml DNaseI, 2.5 mM MgCl_2_, 0.5 mM CaCl_2_) and lysed through several freeze-thaw cycles. Cell debris was pelleted by centrifugation and supernatants transferred to microtitre plates.

The supernatants were then screened for protease inhibition as follows: 10 μl lysate was incubated with 10 μl 50 nM thrombin, 10 μl 100 nM APC or 10 μl 100 nM fXa for 1 h, 20 min or 1 h, respectively. Reactions were stopped by the addition of 100 μl of 0.2 mM S2238 (thrombin), 0.4 mM S2366 (APC) or 0.4 mM S2222 (fXa), and absorbance at 405 nm (A_405_) read. The slope of the plot of A_405_ over time gave an endpoint residual protease activity for the respective protease. Variants that showed low residual APC activity, and therefore high APC inhibitory activity, together with high residual thrombin and/or fXa activity (i.e. low thrombin and/or fXa inhibitory activity) were potential candidates for APC-specific serpins.

Colonies of interest were picked from the archive plates and re-expressed and re-assayed using the method described above. Colonies that were identified in this rescreen as being of interest were then picked again from the archive plates and sequenced at Source Bioscience (Cambridge) using Sanger sequencing.

### Purification of α_1_AT variants for further characterisation

α_1_AT variants were generated by site-directed mutagenesis of the pETSUMO full-length PRS α_1_AT C232S background with the additional E1S mutation and expressed and purified as described previously^23^. Briefly, after overnight expression in Rosetta2(DE3)pLysS, α_1_AT was purified from cell lysate using first a HiTrap IMAC HP column (GE Healthcare), charged with NiSO_4_, followed by further purification on a HiTrap Q Sepharose HP column (GE Healthcare). The SUMO tag was removed by overnight cleavage with SUMO protease and α_1_AT with the tag removed repurified using IMAC and Q Sepharose columns in tandem. Purity of all preparations was assessed by SDS-PAGE. All serpins in this study are referred to by their P2-P1′ sequences e.g. PRS α_1_AT has a P2 Pro, P1 Arg and P1′ Ser.

### Determination of inhibition rate constants using the discontinuous method

Proteases, serpins and substrates were diluted from stock solutions into either thrombin assay buffer (20 mM Tris pH 7.4, 100 mM NaCl, 0.2% w/v BSA, 0.1% w/v PEG 8000) or APC assay buffer for reactions with APC or fXa (20 mM Tris pH 7.4, 100 mM NaCl, 0.2% w/v BSA, 0.1% w/v PEG 8000, 2.5 mM CaCl_2_).

Second-order rate constants (*k*_2_) were measured under pseudo-first order conditions, using at least a 5-fold molar excess of serpin over protease as described^35^. In a non-binding 96-well plate, serpin was mixed with protease and incubated for varying amounts of time. After incubation, the reaction was stopped by the addition of 100 μl 0.4 mM chromogenic substrate appropriate for the respective reaction (S2238 for thrombin, S2366 for APC and S2222 for fXa). Abs_405_ was then read for an appropriate length of time, depending on protease concentration and reactivity with the substrate. The slope of the linear part of the graph plotting A_405_ *versus* time is proportional to the residual protease activity and therefore the residual protease concentration. The negative slope of a plot of the natural logarithm of residual protease activity *versus* incubation time with serpin is the apparent first-order rate constant *k*_obs_. *k*_obs_ was measured for at least six different serpin concentrations and plotted against serpin concentration. Linear regression was used to determine the slope of the plot of *k*_obs_ *versus* serpin concentration. This slope is the second-order rate constant *k*_2_, reported with the standard error of the slope. No statistical analysis was performed, since only substantially altered rate constants relative to the parent sequence were considered.

For each serpin, concentrations were chosen appropriately for the reaction rates. Protease and serpin concentration ranges for the respective reactions were as follows: 0.25–5 μM serpin with 12.5–25 nM human plasma α-thrombin; 0.125–4 μM serpin with 12.5–50 nM human plasma APC; 0.25–2 μM serpin with 12.5–25 nM human plasma fXa.

### Activated partial thromboplastin time (aPTT)

Normal plasma from three different individuals was mixed to generate pooled plasma. 50 μl plasma was mixed with 50 μl serpin diluted to 30 μM in TBS (final reaction concentration 5 μM). 100 μl aPTT reagent (Triniclot automated aPTT reagent) was added and the reaction incubated for 5 min. 100 μl 25 mM CaCl_2_ was added to start the clotting reaction. Time to clot formation was measured on a Stago Start coagulation analyser. All reagents were warmed to 37 °C prior to start of the reaction and reactions were carried out at 37 °C. Where an error is given, the value is the average of at least three measurements and the error is the standard deviation.
